# Glomerular hyperfiltration defined by eGFR and long-term clinical outcomes: a systematic review and meta-analysis

**DOI:** 10.3389/fmed.2025.1714170

**Published:** 2026-01-13

**Authors:** Qinbo Yang, Kaixin Lei, Peiyan Sun, Ao Chen, Baihai Su

**Affiliations:** 1 Department of Nephrology, Kidney Research Institute, West China Hospital of Sichuan University, Chengdu, Sichuan, China; 2 West China Hospital, West China School of Medicine, Sichuan University, Chengdu, China

**Keywords:** glomerular hyperfiltration, GHF, eGFR, albuminuria, cardiovascular disease, mortality, meta-analysis

## Abstract

**Background:**

Glomerular hyperfiltration (GHF) has been widely recognized as a potential risk factor. However, current evidence regarding both its precise definition and clinical prognostic value remains inconclusive.

**Methods:**

We systematically searched PubMed, Embase, and the Cochrane Library, as well as reference lists of relevant articles. Cohort studies examining the association between eGFR-defined GHF and clinical outcomes were included, and meta-analyses were conducted using a random-effects model. Cross-sectional studies and data from the National Health and Nutrition Examination Survey (NHANES) were used to estimate and compare the 95th percentile of eGFR in healthy populations in China and the United States.

**Results:**

The 95th percentile eGFR values among healthy individuals ranged from 94.7 to 146.7 mL/min/1.73 m^2^. A total of 17 studies, including 11,563,332 participants, were included in the systematic review. GHF was significantly associated with an increased risk of all-cause mortality (HR, 1.30; 95% CI, 1.18–1.42) and incident albuminuria (HR, 1.43; 95% CI, 1.05–1.93) but was not significantly associated with cardiovascular disease. In addition, individual studies suggested potential associations between GHF and rapid eGFR decline, the incident dementia, and the incident non-alcoholic fatty liver disease.

**Conclusion:**

Although eGFR is subject to measurement biases in non-CKD populations, extremely high values may still indicate underlying risks not captured by routine monitoring. This study provides reference values for the 95th percentile of eGFR in the general Chinese and American populations and underscores the need to validate individualized definitions of GHF. Given the limited and heterogeneous evidence base, these findings should be interpreted cautiously, and further prospective studies are needed to clarify the clinical significance of GHF.

**Systematic review registration:**

INPLASY 202540039.

## 1 Introduction

A low estimated glomerular filtration rate (eGFR) and chronic kidney disease (CKD) are well-established risk factors for cardiovascular disease (CVD) and mortality. In contrast, glomerular hyperfiltration (GHF) represents the opposite end of the renal function spectrum. GHF has been observed not only in individuals with established kidney disease but also in healthy populations and in those with obesity or diabetes (1). For example, the prevalence of GHF in patients with early-stage type 2 diabetes has been reported to range from 6% to 73% (2).

These findings suggest that GHF may represent a preclinical phase of diabetic nephropathy and that the association between eGFR and adverse outcomes often follows a J- or U-shaped pattern, with elevated risks at both reduced and abnormally high levels (3–6). However, the definition of GHF remains heterogeneous, most commonly based on an eGFR exceeding two standard deviations above the age- and sex-specific mean, although thresholds vary substantially across populations (1). Despite growing interest in eGFR-defined GHF, a systematic synthesis of the evidence is still lacking. Large-scale cross-sectional studies have examined eGFR distributions in healthy individuals, providing valuable reference data for defining the 95th percentile thresholds (7–9). Against this background, we conducted a systematic review and meta-analysis of cohort studies to assess the association between eGFR-defined GHF and clinical outcomes, and to quantitatively establish age- and sex-specific 95th-percentile eGFR values in the healthy population.

## 2 Methods

The protocol of this systematic review and meta-analysis has registered with International Platform of Registered Systematic Review and Meta-analysis Protocols (INPLASY 202540039) and reported following the Preferred Reporting Items for Systematic Reviews and Meta-Analyses (PRISMA) 2020 statement (10).

### 2.1 Search strategy and eligibility criteria

We systematically searched PubMed, Embase, and the Cochrane Library for studies on associations between GHF and long-term clinical outcomes published from January 2014 to March 2025. Studies published before 2014 were identified through citation tracking (11), and detailed search strategies are provided in Supplementary methods.

Two researchers (Q.Y. and K.L.) independently screened the literature. Eligible studies met the following criteria: (1) cohort design; (2) GHF was defined on the basis of eGFR, with no restriction on the equation used for estimation; (3) GHF as the exposure of interest; and (4) multivariable-adjusted odds ratios (ORs) or hazard ratios (HRs) with 95% confidence intervals (CIs) were reported. In the included studies, statistical models were required to adjust for at least the key confounders. The key confounders were selected based on prior literature as potential causal factors linking from GHF to mortality, and included sex, age, smoking status, body mass index (or equivalent measures), blood pressure or history of hypertension, and HbA1c or diabetes (2, 12, 13). We excluded conference abstracts, cross-sectional studies, reviews, non-English publications, and animal studies. Also excluded were studies using inulin or iohexol clearance, and those involving pregnant women or juveniles.

### 2.2 Outcomes

Primary outcomes were all-cause mortality, cardiovascular disease (CVD) events, and two kidney-related outcomes (albuminuria and rapid eGFR decline). Additionally, 30-day major adverse events were assessed in post-operative patients. CVD events included composite outcomes such as stroke, myocardial infarction, and heart failure requiring hospitalization. Albuminuria was defined as albumin excretion ≥30 mg/24 h. Rapid eGFR decline was defined as a drop >3 mL/min/1.73 m^2^/year. In post-operative populations, 30-day adverse events included reoperation, cardiac arrest with requiring cardiopulmonary resuscitation, myocardial infarction, stroke, or death. Studies not aligned with our population or outcomes were reviewed systematically but excluded from meta-analysis.

### 2.3 Data extraction

Before data extraction, all four reviewers (Q.Y., K.L., P.S. and A.C.) underwent joint training and conducted pilot exercises. Each reviewer then independently extracted data using a standardized form. Extracted information included study author, year of publication, sample size, length of follow-up, eGFR equations, definitions of GHF, reference group, adjusted confounders, outcomes, and effect estimates. Fully adjusted odds ratios (ORs) or hazard ratios (HRs) with corresponding 95% confidence intervals (CIs) were prioritized. When estimates were available only in graphical form, values were extracted using WebPlotDigitizer (14, 15). For studies with multiple publications based on the same cohort, data were extracted from the report with the largest sample size or the longest follow-up duration.

### 2.4 Quality assessment

Study quality was assessed using the Newcastle–Ottawa Scale (NOS), which covers selection, comparability, and outcome/exposure assessment. Studies scoring ≥6 (out of 9) were deemed high quality (16). Two reviewers (K.L. and Q.Y.) independently rated all studies. Disagreements were resolved by discussion; unresolved cases were reviewed by a third researcher (B.S.) to reach consensus.

### 2.5 Data synthesis and meta-analysis

Study populations were classified into GHF and non-GHF groups according to each study's original criteria. For each outcome, hazard ratios (HRs) or odds ratios (ORs) with 95% confidence intervals (CIs) were extracted and log-transformed with their standard errors to stabilize variance and approximate normality. Pooled estimates were calculated using random-effects models with inverse variance weighting, and the results with 95% CIs were back-transformed and presented as forest plots. For studies reporting multiple subgroup estimates, subgroup results were pooled within study to generate a single effect size for the overall meta-analysis. In sensitivity analyses, subgroup estimates were retained and analyzed using multilevel random-effects models to account for within- and between-study heterogeneity.

Heterogeneity across the studies was evaluated with the τ^2^ and Cochran's Q test. A *P*-value < 0.1 in the Q test was considered statistically significant, while an *I*^2^ ≥ 50% indicated substantial heterogeneity (17). Publication bias was assessed for outcomes with data from at least five studies. Funnel plots were visually inspected for asymmetry, and Egger's test was applied to statistically evaluate potential publication bias (18). Subgroup analyses were performed across different populations and eGFR equations. Sensitivity analyses were performed using a leave-one-out approach to assess the robustness of the results. In addition, meta-regression analyses were undertaken to examine whether follow-up duration contributed to between-study heterogeneity. All analyses were conducted using the *meta* package (19).

Additionally, we applied data from National Health and Nutrition Examination Survey (NHANES) and Chinese cross-sectional studies to evaluate the 95th percentile values and their 95%CIs in general individuals by using Bootstrap Method (Supplementary methods). After exclusion of patients who have hypertension, obesity (body mass index ≥ 30 kg/m^2^), diabetes, chronic kidney disease (eGFR < 60 mL/min/1.73 m^2^ or urine albuminuria-to-creatinine ratio ≥30 mg/g) and cardiovascular disease (any report of myocardial infraction, stroke, heart disease, coronary heart disease or angina pectoris), a total of 15,129 participants were enrolled (Supplementary Figure S1). Bootstrap analysis leveraged the *boot* package in R software and created a random seed to facilitate the bootstrap process, wherein one-third of the sample size of the target population was drawn per iteration, with a total of 5,000 iterations performed. All statistical analysis were finished via R software (Version 4.2.2).

## 3 Results

### 3.1 Characteristics of included studies

**Figure 1:**
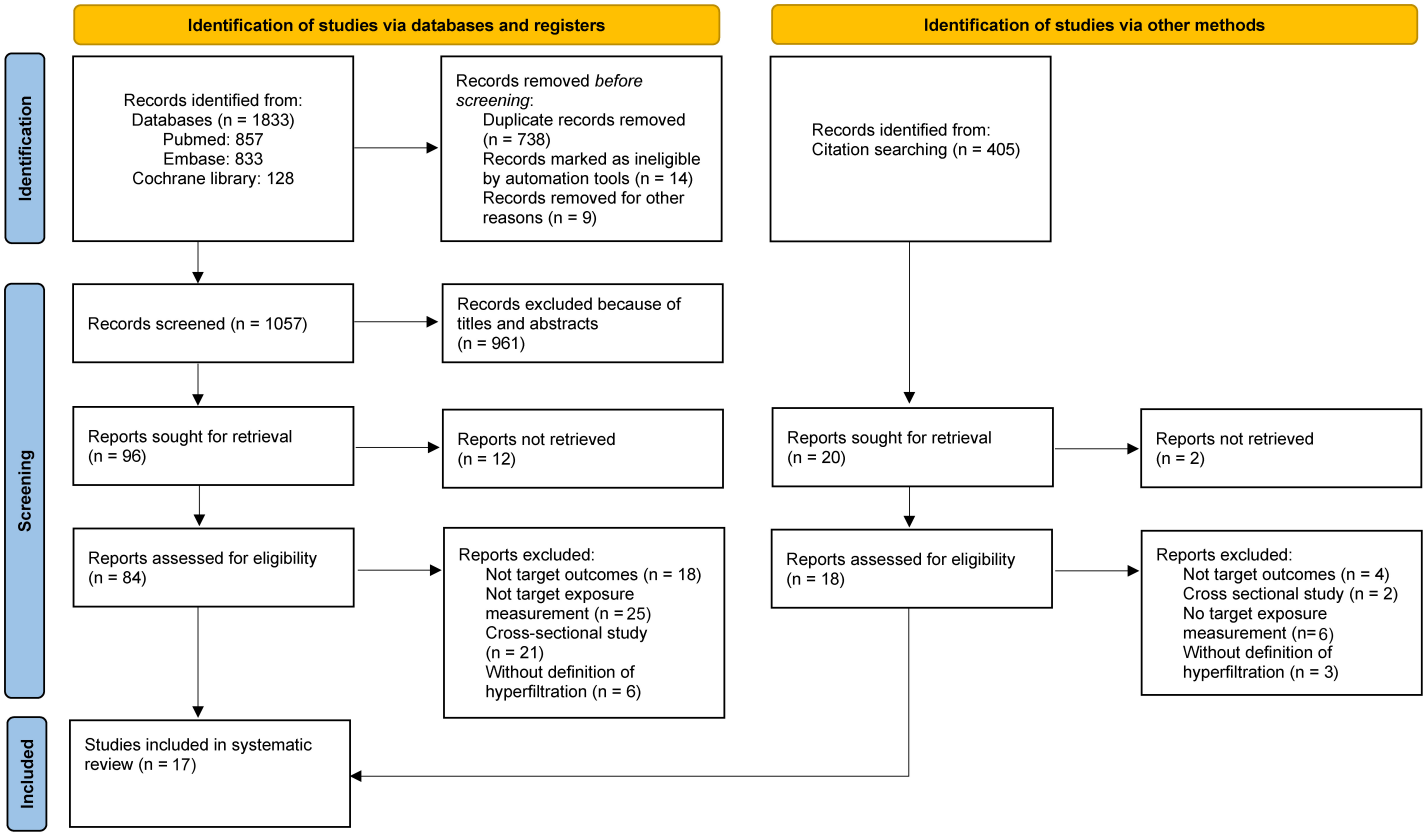
Preferred reporting items for systematic reviews and meta-analyses flow diagram.

As illustrated in Figure 1, the screening and selection process is summarized. A total of 1,833 records were identified from electronic databases and 405 additional records through citation searching (11). After rigorous screening and full-text review, 17 studies were finally included.

The main characteristics of the included studies are summarized in Table 1. Collectively, they enrolled 11,563,332 participants, including community-based cohorts, health screening cohorts, individuals with type 2 diabetes, and populations at cardiovascular risk. In most studies, GHF was defined using age- or sex-specific eGFR percentiles, while the reference groups were defined as participants with eGFR < 60 or < 90 mL/min/1.73 m^2^, or those with mid-range eGFR values. Follow-up duration ranged from 3 to 15 years. Seven studies, including a total of 743,907 participants, investigated the association between GHF and all-cause mortality. Three studies, involving 2,015,560 participants, reported on cardiovascular disease (CVD) events. Another three studies focused on kidney-related outcomes, while two investigated post-operative adverse events in the post-operative population. Additionally, four studies separately investigated the associations of GHF with various outcomes, such as atrial fibrillation, incident dementia, new-onset cancer, and incident non-alcoholic fatty liver disease (NAFLD).

**Table 1:** Baseline characteristics of included studies.

**Author, year**	**Country**	**Population**	**Sample sizes**	**Follow-up duration**	**eGFR measurement**	**Definition of hyperfiltration**	**Non-exposed cohorts**	**Outcomes**
Dupuis et al., 2020 (34)	Canada	Middle-aged population in Canada	20,004	6 years	CKD-EPI	eGFR > 95th percentile (age- and sex-stratified)	eGFR between 25th and 75th percentile	CVD
Kang et al., 2023 (24)	Korean	Individuals underwent health screenings	2,645,042	10 years	CKD-EPI	eGFR > 120 mL/min/1.73 m^2^	60 ≤ eGFR < 90	Atrial fibrillation
Kang et al., 2020 (21)	Korean	Community dwellers	2,244,582	3 years	CKD-EPI	eGFR > 95th percentile (age- and sex-stratified)	eGFR between 25th and 75th percentile	Incident dementia
Kim et al., 2020 (22)	Korean	Individuals aged over 18 years	1,953,123	4.4 years	CKD-EPI	eGFR > 95th percentile (age- and sex-stratified)	eGFR between 35th and 50th percentile	Incident cancer
Kim et al., 2021 (35)	Korean	Individuals aged over 45 years	565,902	3 years	CKD-EPI	eGFR > 95^th^ percentile (age-stratified)	eGFR between 40th to 59th percentile	All-cause mortality
Koo et al., 2022 (23)	Korean	Adults aged 20 to 65 years	147,162	7 years	CKD-EPI	eGFR > 95th percentile (age- and sex-stratified)	eGFR between 50th and 64th percentile	Incident NFALD
Oh et al., 2020 (36)	Korean	Individuals aged over 20 years	19,046	13 years	CKD-EPI	eGFR > 97.5^th^ percentile (age- and sex-stratified)	eGFR < 95th percentile	Incident albuminuria: progression of eGFR decline
Park et al., 2015 (37)	Korean	Individuals aged over 20 years	43,503	17 years	CKD-EPI	eGFR > 95th percentile (age- and sex-stratified)	eGFR between 60th and 95th percentile	All-cause and cause specific mortality; CVD; incident hypertension; incident diabetes; incident albuminuria
Yoo et al., 2017 (38)	Korean	Individuals aged 40 to 80 years	114,966	75 months	CKD-EPI	eGFR > 95th percentile (age- and sex-stratified)	eGFR < 95th percentile	All-cause mortality
Korhonen et al., 2023 (39)	Finland	Individuals with cardiovascular risks	1,747	14 years	CKD-EPI	eGFR > 132 mL/min/1.73 m^2^	85 ≤ eGFR < 113	All-cause mortality
Mostofsky et al., 2009 (40)	U.S.	Patients with history of TIA or ischemic stroke	1,175	8 years	MDRD	eGFR > 125 mL/min/1.73 m^2^	75 ≤ eGFR < 125	All-cause mortality
Putaala et al., 2011 (41)	Finland	Patients with first-ever ischemic stroke	958	16 years	MDRD	eGFR > 120 mL/min/1.73 m^2^	60 ≤ eGFR < 120	All-cause mortality
Moriya et al., 2017 (42)	Japan	Patients with type 2 diabetes or HbA1c over 6.5%	1,047	8 years	MDRD	eGFR > 120 mL/min/1.73 m^2^	90 ≤ eGFR < 120	Rapid eGFR decline
Penno et al., 2020 (43)	Italy	Patients with type 2 diabetes	15,656	2 years	CKD-EPI	eGFR > 95th percentile (age- and sex-stratified)	eGFR between 5th and 95th percentile	All-cause mortality
Chung et al., 2025 (44)	Korean	Korean people with type 2 diabetes	1,952,053	7 years	CKD-EPI	eGFR > 95th percentile (age- and sex-stratified)	eGFR between 40th and 60th percentile	CVD
Carlos et al., 2023 (45)	U.S.	Adult patients undergone surgical procedures	1,668,447	30 days	CKD-EPI	eGFR > 95th percentile (age- and sex-stratified)	eGFR between 5th and 95th percentile	Postoperative 30-day major adverse outcomes
Kavin et al., 2021 (46)	U.S.	Adult patients undergone surgical procedures	168,919	30 days	MDRD	eGFR > 120 mL/min/1.73 m^2^	90 ≤ eGFR < 120	Postoperative 30-day major adverse outcomes

CVD, cardiovascular disease; ESRD, end-stage renal disease; CKD-EPI, Chronic Kidney Disease Epidemiology Collaboration; MDRD, the Modification of Diet in Renal Disease variable formula; NFALD, non-alcoholic fatty liver disease.

### 3.2 Evaluation of 95th percentile eGFR values

**Figure 2:**
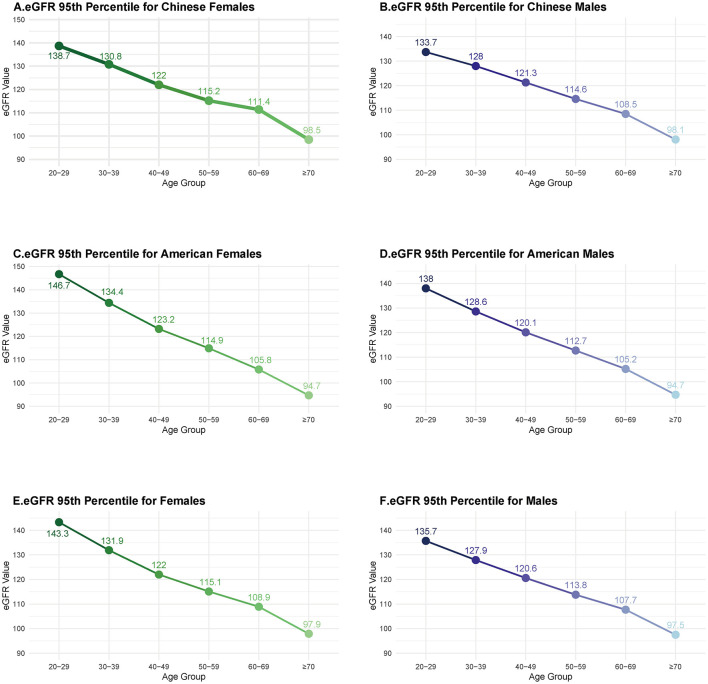
95th percentiles of eGFR stratified by age, sex, and region. **(A)** Chinese females; **(B)** Chinese males; **(C)** American males; **(D)** American females; **(E)** combined data: Chinese and American females; **(F)** combined data: Chinese and American males.

In this section, data from two large-scale cross-sectional studies in China (39,623 healthy individuals) (7, 20), together with data from 15,219 NHANES participants, were used to examine the distribution of eGFR (Supplementary Figure S1). In all populations, eGFR was estimated using the CKD-EPI equation. The mean 95th percentile eGFR values across age- and sex-specific strata, derived from 5,000 bootstrap iterations, are shown in Figure 2, with corresponding 95% confidence intervals in Supplementary Table S1. As illustrated, the 95th percentile declined progressively with age, and women consistently exhibited higher values than men. For example, among individuals aged 50–59 years in China, the 95th percentile was 115.2 mL/min/1.73 m^2^ in women compared with 114.6 mL/min/1.73 m^2^ in men. In participants younger than 50 years, the 95th percentile exceeded 120 mL/min/1.73 m^2^, peaking at 146.7 mL/min/1.73 m^2^ in American women and 138.7 mL/min/1.73 m^2^ in Chinese women. Notably, the American population exhibited systematically higher 95th percentile values than their Chinese counterparts before age 60, whereas the Chinese population modestly exceeded the American values thereafter.

### 3.3 Quality assessments

The details of the quality assessment are provided in Supplementary Table S2. Of the 13 studies included in the effect-size pooling, 11 were rated as high quality, while 2 were judged to raise some concerns. In these lower-rated studies, the primary source of potential bias was related to the selection domain.

### 3.4 Meta-analysis for outcomes

**Figure 3:**
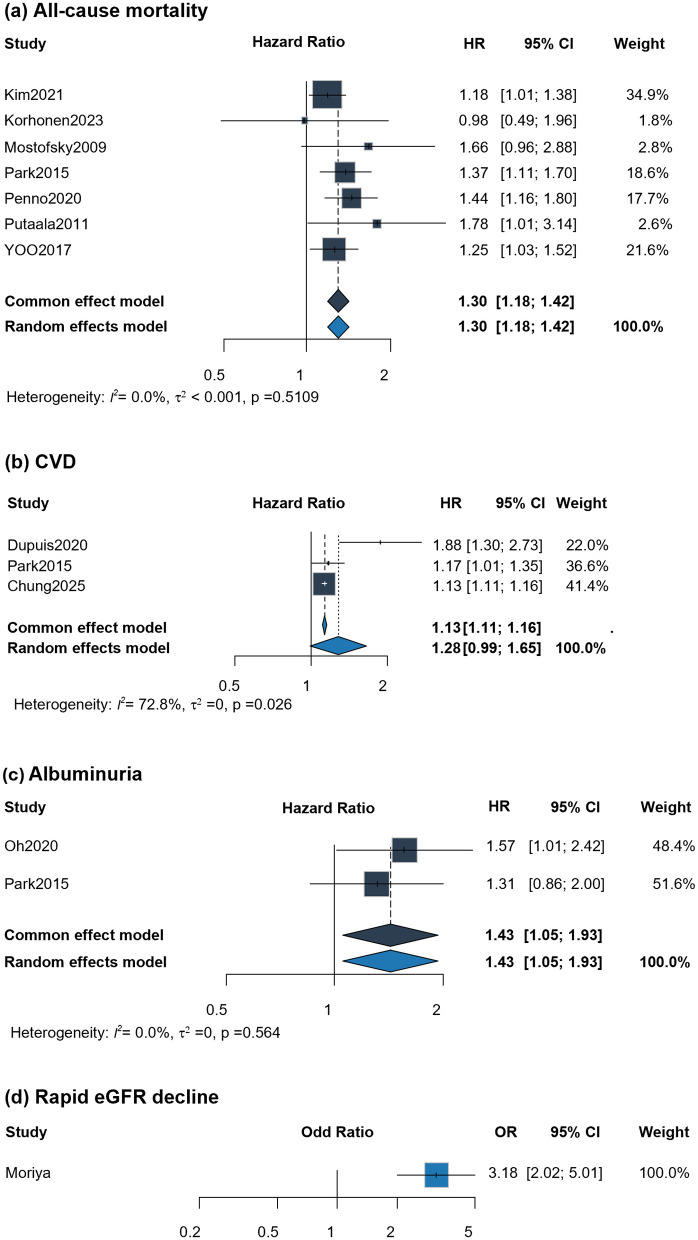
Forest plots on major study outcomes. **(a)** All-cause mortality; **(b)** CVD; **(c)** Incident albuminuria; **(d)** Rapid eGFR decline.

The meta-analysis demonstrated that GHF was significantly linked to an increased risk of mortality (HR, 1.30; 95% CI, 1.18–1.42). Among the seven studies evaluating all-cause mortality, six were rated as high quality and together contributed 97.2% of the weight in the pooled hazard ratio (Figure 3). Between-study heterogeneity was negligible (*I*^2^ = 0%, *p* = 0.511), and Egger's test provided no evidence of publication bias (*p* = 0.336; Supplementary Figure S2). As shown in Supplementary Table S3, the elevated mortality risk was consistent across subgroups, remaining evident in the general population (HR, 1.22; 95% CI, 1.09–1.36) as well as in individuals with cardiovascular risk factors (HR, 1.50; 95% CI, 1.06–2.11). Similar consistency was observed across studies using different eGFR equations.

Only three studies reported on the association between GHF and CVD events. The available evidence does not demonstrate a statistically significant association between GHF and CVD events (HR, 1.28; 95% CI, 0.99–1.65). All three studies included in the analysis were rated as high quality, although heterogeneity was substantial (*I*^2^ = 72.8%, *p* = 0.026). In the general population subgroup, the association likewise did not reach statistical significance (Supplementary Table S3). Two studies reported on the incident albuminuria. One high-quality study contributed 51.6% of the overall weight. The pooled analysis demonstrated a significant association between GHF and the incident albuminuria (HR, 1.43; 95% CI, 1.05–1.93). Both studies enrolled Korean health checkup populations over 20 years of age, although from different time periods. In addition, one study in individuals with type 2 diabetes found that those with GHF had a higher risk of rapid eGFR decline (OR, 3.18; 95% CI, 2.02–5.01).

Beyond mortality, CVD, and kidney outcomes, several studies explored broader clinical endpoints. GHF was linked to greater risks of dementia (HR, 1.09; 95% CI, 1.03–1.15) (21), cancer (HR, 1.08; 95% CI, 1.04–1.13) (22) and nonalcoholic fatty liver disease (HR, 1.21; 95% CI, 1.14–1.29) (23). By contrast, it appeared protective against atrial fibrillation (HR, 0.88; 95% CI, 0.78–0.98) (24). In the post-operative population, two studies assessed 30-day major adverse events, but no statistically significant relationship with GHF was detected (Supplementary Figure S3).

### 3.5 Sensitivity analysis and meta-regression

**Figure 4:**
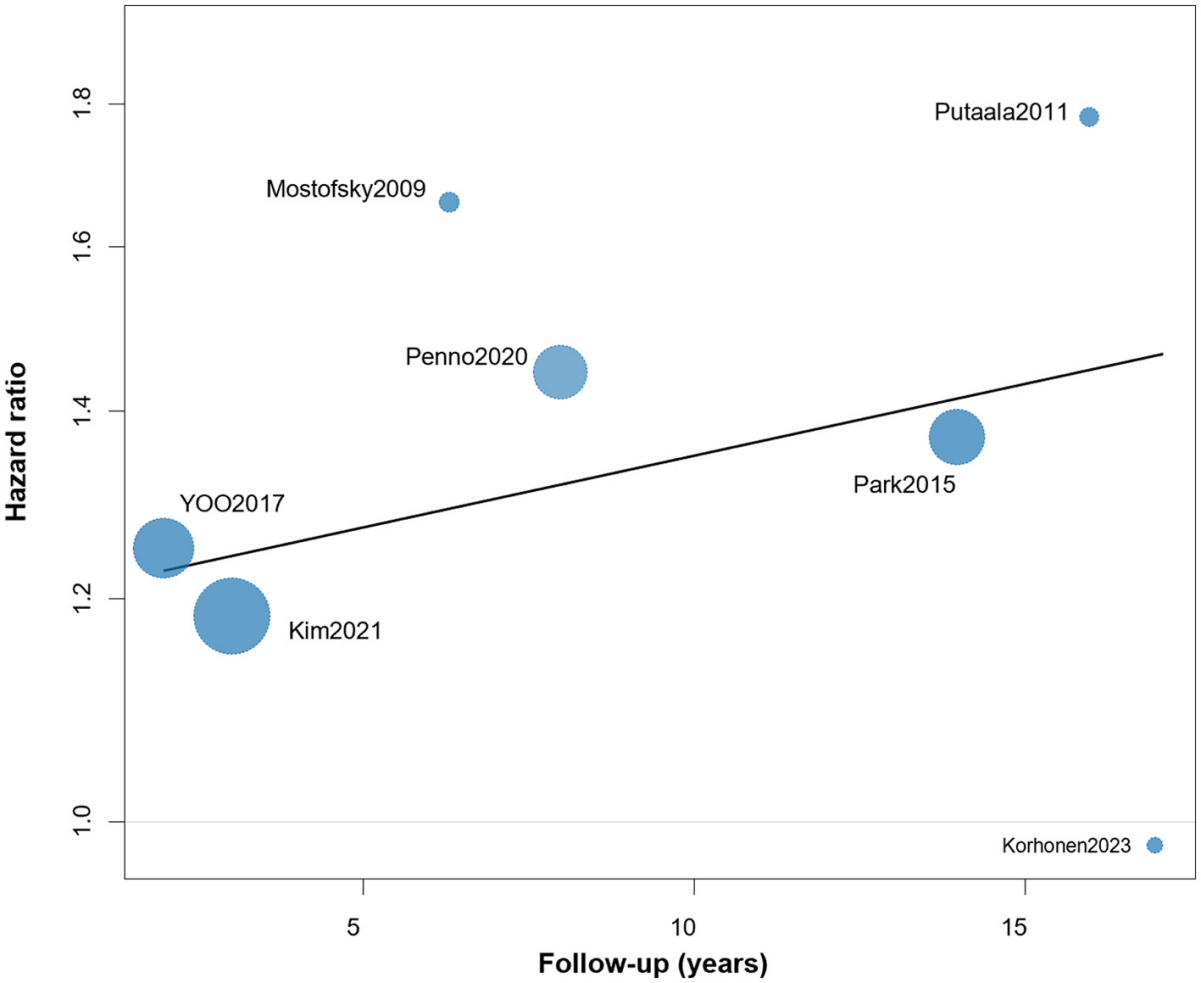
Meta-regression analysis of follow-up duration for all-cause mortality.

Sensitivity analyses using a leave-one-out approach showed that the pooled effect estimates for all-cause mortality remained essentially unchanged after sequentially omitting each study (Supplementary Table S4). Multilevel random-effects models likewise confirmed the robustness of the association with all-cause mortality (Supplementary Figure S4). In addition, a meta-regression analysis was conducted to examine whether follow-up duration accounted for variation in effect sizes (Figure 4), but no significant association was observed (*p* > 0.05).

## 4 Discussion

GHF reflects an increase in single-nephron GFR (SNGFR) driven primarily by hemodynamic alterations (1). A variety of factors can lead to decreased pre-glomerular resistance, increased post-glomerular resistance, or impaired tubuloglomerular feedback. These changes increase glomerular capillary hydraulic pressure, which subsequently raises SNGFR and results in sustaining hyperfiltration. Persistent biomechanical stress from chronic GHF drives progressive structural injury. Elevated hydraulic and shear forces deform the capillary wall, enlarge the glomerular basement membrane, and elongate podocyte foot processes. Over time, declining permeability, disruption of podocyte junctions, and eventual foot process effacement emerge. As podocytes have limited regenerative capacity, continued stress leads to podocyte loss, impaired filtration, glomerulosclerosis, and progressive GFR decline (25, 26).

Although its definition remains debated, most studies define GHF as an eGFR exceeding the 95th percentile of the population distribution (1). Eriksen et al. reported that, in a general population aged 50–64 years without baseline CVD, diabetes, or kidney disease, elevated iohexol clearance was associated with a lower risk of CVD but not with all-cause mortality; in contrast, elevated eGFR showed no association with CVD but was associated with an increased risk of all-cause mortality (27). These findings regarding elevated eGFR are consistent with the conclusions on GHF in our study. Although results based on iohexol clearance and eGFR do not fully align, this does not negate the potential role of eGFR. The major concern always lies in the limited accuracy of eGFR in certain populations—such as healthy individuals, patients with early-stage diabetes, or those with low muscle mass—due to inherent biases in eGFR equations (28). Notably, our aim is not to assess the precision of eGFR as a marker of true glomerular filtration. eGFR underestimates measured GFR when the latter lies within the normal range, but overestimates it in individuals with reduced muscle mass, malnutrition, or cachexia (29, 30). These systematic, bidirectional biases suggest that markedly elevated eGFR values in populations may capture multiple underlying risks that are not monitored in routine clinical monitoring. Given its accessibility and cost-effectiveness, eGFR is particularly appropriate for population-based screening and long-term monitoring. Therefore, we restricted our analysis to studies in which GHF was defined on the basis of eGFR.

Considering that both eGFR and GHF may be influenced by multiple factors, we included only studies that reported risk estimates adjusted for relevant confounders. Across these studies, GHF defined by baseline eGFR was consistently associated with an increased risk of all-cause mortality. However, the current evidence does not support a statistically significant association between GHF and cardiovascular disease. In addition, although limited evidence suggests that GHF may be associated with incident albuminuria in healthy populations and with accelerated eGFR decline in individuals with diabetes, these findings should be interpreted with caution given the limited or heterogeneous evidence base. Similarly, the individually reported associations with renal function decline, incident dementia, and non-alcoholic fatty liver disease should be considered preliminary.

To our knowledge, this is the first study to directly compare the 95th percentile values of eGFR across different regions. Megan et al. assessed eGFR distributions using various equations in healthy individuals. Under the CKD-EPI equation, the 95th percentile values in healthy European populations were nearly identical to those reported in NHANES data (Figure 2), which is consistent with the similar racial composition of these cohorts (9). However, among adults over 60 years of age, Chinese participants exhibited higher 95th percentile values compared with their American counterparts. This difference may reflect ethnic variability in age-related renal decline, as previous studies have documented more rapid eGFR loss among Black and Hispanic populations (31). While further validation is needed in larger cohorts, our findings highlight considerable variability in eGFR by age, sex, and race, which increases the complexity and uncertainty in establishing a universal definition of GHF. In addition, our results can be used as reference values for defining GHF across age and sex groups in the Chinese population.

Beyond differences in race, sex, and age, several studies have suggested that the association between GHF and adverse outcomes may be modified by proteinuria, with significant risks observed only in the presence of high levels of proteinuria (27, 32). These findings highlight the importance of considering population characteristics and clinical context when analyzing an eGFR-based definition of GHF. A critical limitation is that nearly all available studies relied on a single eGFR measurement to define GHF, overlooking the physiological variability and dynamic changes of glomerular filtration. In a hypertensive cohort with a median follow-up of 8.5 years, the highest risk of developing albuminuria was observed among participants who initially presented with GHF but reverted to normal filtration, rather than among those who maintained persistent GHF (33). In early stages of diabetes or hypertension, nephron loss may already be present; however, compensatory hyperfiltration in the remaining nephrons can maintain an overall eGFR within the normal range (1). This indicates that a normal eGFR value does not exclude the presence of compensatory elevated SNGFR. Therefore, applying the same threshold across populations with different disease states may not be optimal. We advocate for longitudinal studies with repeated eGFR assessments to capture individual trajectories more precisely. Such an approach would help identify within-person relative elevations in eGFR that might otherwise be overlooked.

The present study has several limitations, largely determined by the quality and availability of existing evidence. First, our findings were based on a limited number of studies, although the individual cohorts generally included large sample sizes, which may affect the reliability of our conclusions. Second, substantial heterogeneity remained, particularly in analyses of CVD outcomes. This may stem from differences in study populations and variations in the definitions of non-exposed groups. Third, the limited populations restricts the generalizability of our findings. As many of the included studies were conducted in diverse populations from Korea, the results may not be readily generalized to other populations or ethnic groups. Fourth, while subgroup analysis showed stable results across populations, considering the variation in nephron count and disease risk in different cohorts, the relationship between GHF and mortality risk in these varied populations needs to be validated in future studies. Finally, selection bias may have arisen from excluding non-English publications and studies with incomplete adjustment for confounders. These limitations should be taken into account when interpreting the results.

## 5 Conclusion

This systematic review and meta-analysis indicate that eGFR-defined GHF is associated with a higher risk of all-cause mortality. Although no statistically significant association was observed between GHF and cardiovascular disease, the small number of available studies and the significant heterogeneity warrant cautious interpretation. We also observed substantial variation in the 95th percentile of eGFR across age, sex, and ethnic groups, underscoring the need for more refined and individualized definitions. Future research should prioritize the development of individual-specific criteria for GHF and investigate whether interventions aimed at lowering intraglomerular pressure—such as SGLT2 inhibitors or RAAS blockade—can mitigate the associated risks.

## Data Availability

The original contributions presented in the study are included in the article/Supplementary material, further inquiries can be directed to the corresponding author.
