# Supplementary material for: Glomerular hyperfiltration defined by eGFR and long-term clinical outcomes: a systematic review and meta-analysis
Source: Front Med (Lausanne). 2026 Jan 13;12:1714170. doi: 10.3389/fmed.2025.1714170 (PMC12834743; doi:10.3389/fmed.2025.1714170)
Supplement: Supplementary file 1 [file Supplementary_file_1.docx]

**Supplement Methods**

1. Details on search strategies

As this systematic review and meta-analysis did not included restriction for population, the key term for search is “hyperfiltrating” or “hyperfiltration”. The search queries for three databases as follows (1): (("hyperfiltration"[Title/Abstract] OR "hyperfiltrating"[Title/Abstract]) NOT ("Congress"[Publication Type] OR ("Review"[Publication Type] OR "Review Literature as Topic"[MeSH Terms] OR "Case Reports"[Publication Type]))) AND (2014:2024[pdat]) in Pubmed; (2) (hyperfiltration: ab.ti OR hyperfiltration: ab.ti) AND [2014-2024]/py AND (article/lim OR article in press/lim) in Embase; (3): hyperfiltration OR hyperfiltrating within title and abstract keyword in Cochrane library.

1. Data extraction for estimation of eGFR 95^th^ percentile.

For estimation in Chinese population, two cross-sectional study based on healthy individuals and eGFR calculated by creatinine-based CKD-EPI equation were included^1,2^. Information on age and sex specific groups were extracted for analysis. In terms of American estimates, we utilized data from National Health and Nutrition Examination Survey (NHANES). This data set employed sophisticated sampling methods to ensure representability of general American population^3^. In order to evaluate 95^th^ percentile in healthy population, with reference to European population^4^, patients who have hypertension, obesity (body mass index ≥ 30kg/ m^2^), diabetes, chronic kidney disease (eGFR < 60 mL/min/1.73 m^2^ or urine albuminuria-to-creatinine ratio ≥ 30 mg/g) and cardiovascular disease (reports of myocardial infraction, stroke, heart disease, coronary heart disease or angina pectoris) were excluded (Figure S1). A total of 15129 healthy individuals were enrolled in further analysis based on age and sex-specific categories.

1. Bootstrap analysis

Bootstrap method was employed to estimate 95^th^ percentile of eGFR in age and sex-specific groups (20 - 29, 30 – 39, 40 – 49, 50 – 59, 60 – 69 and ≥ 70 in males or females respectively). Bootstrap is a resampling technique extensively employed in machine learning to estimate the distribution and parameters of a population through repeated sampling with replacement^5^. In this study, we utilized the bootstrap method to derive age- and sex-specific 95th percentiles and their 95% CIs for Chinese, American, and mixed cohorts. By leveraging the "boot" package in R software, we created a random seed to facilitate the bootstrap process, wherein one-third of the sample size of the target population was drawn per iteration, with a total of 5000 iterations performed. The resulting 95th percentile estimations and their associated 95% confidence intervals are detailed in Table S2.

Reference:

1. Yue L, Fan L, Du X. Age- and Sex-Specific Reference Values of Estimated Glomerular Filtration Rate in Chinese Population. *Gerontology*. 2021;67(4):397-402. doi:10.1159/000513451

2. Ma Y, Zhan J, Xu G. Reference values of glomerular filtration rate for healthy adults in southern China: a cross-sectional survey. *Ther Adv Chronic Dis*. 2021;12:20406223211035287. doi:10.1177/20406223211035287

3. Paulose-Ram R, Graber JE, Woodwell D, Ahluwalia N. The National Health and Nutrition Examination Survey (NHANES), 2021-2022: Adapting Data Collection in a COVID-19 Environment. *Am J Public Health*. Dec 2021;111(12):2149-2156. doi:10.2105/ajph.2021.306517

4. Astley ME, Chesnaye NC, Hallan S, et al. Age- and sex-specific reference values of estimated glomerular filtration rate for European adults. *Kidney Int*. Mar 22 2025;doi:10.1016/j.kint.2025.02.025

5. Kaity S, Sah SK, Karanwad T, Banerjee S. Bootstrap Statistics and Its Application in Disintegration and Dissolution Data Analysis. *Mol Pharm*. Aug 7 2023;20(8):3791-3803. doi:10.1021/acs.molpharmaceut.3c00222

**Supplement Tables and Figures**

Table S1 Reference eGFR value of hyperfiltration group in healthy population.

| **Male** |  |  |  | **Female** |  |  |  |
| --- | --- | --- | --- | --- | --- | --- | --- |
| Age groups | Estimated 95^th^ percentile | Lower 95%CI | Upper 95% CI | Age groups | Estimated 95^th^ percentile | Lower 95%CI | Upper 95% CI |
| **Chinese individuals** |  |  |  |  |  |  |  |
| 20-29 | 133.7 | 132.5 | 134.8 | 20-29 | 138.7 | 137.5 | 140 |
| 30-39 | 128 | 126.9 | 129.1 | 30-39 | 130.8 | 130 | 131.5 |
| 40-49 | 121.3 | 120.1 | 122.2 | 40-49 | 122 | 121.1 | 122.8 |
| 50-59 | 114.6 | 113.4 | 115.6 | 50-59 | 115.2 | 113.8 | 116.3 |
| 60-69 | 108.5 | 107.1 | 109.8 | 60-69 | 111.4 | 108.8 | 113.7 |
| ≥70 | 98.1 | 97.2 | 99.3 | ≥70 | 98.5 | 97.3 | 99.7 |
| **American individuals** |  |  |  |  |  |  |  |
| 20-29 | 138 | 136.9 | 139.2 | 20-29 | 146.7 | 145.7 | 147.8 |
| 30-39 | 128.6 | 127.1 | 129.9 | 30-39 | 134.4 | 133.3 | 135.5 |
| 40-49 | 120.1 | 118.5 | 121.4 | 40-49 | 123.2 | 121.6 | 124.3 |
| 50-59 | 112.7 | 111.1 | 114.4 | 50-59 | 114.9 | 113.7 | 117 |
| 60-69 | 105.2 | 102.9 | 106.7 | 60-69 | 105.8 | 103.5 | 107.6 |
| ≥70 | 94.7 | 92.9 | 96.3 | ≥70 | 94.7 | 92.4 | 96.7 |
| **The whole population** |  |  |  |  |  |  |  |
| 20-29 | 135.7 | 134.8 | 136.5 | 20-29 | 143.3 | 142.3 | 144.3 |
| 30-39 | 127.9 | 127.1 | 128.9 | 30-39 | 131.9 | 131.2 | 132.5 |
| 40-49 | 120.6 | 119.7 | 121.5 | 40-49 | 122 | 121.2 | 122.7 |
| 50-59 | 113.8 | 112.8 | 114.8 | 50-59 | 115.1 | 114.1 | 116.1 |
| 60-69 | 107.7 | 106.7 | 108.8 | 60-69 | 108.9 | 106.9 | 110.8 |
| ≥70 | 97.5 | 96.5 | 98.5 | ≥70 | 97.9 | 96.9 | 99.2 |

Table S2 Details in Newcastle-Ottawa Scale for studies qualified for meta-analysis.

| **Study ID** | **Selection (4 points)** | | | | **Comparability (2 points)** | **Outcome (3 points)** | | | **Total** |
| --- | --- | --- | --- | --- | --- | --- | --- | --- | --- |
|  | Representativeness of study population | Selection of non-exposed cohort | Ascertainment of exposure | Outcome do not occur at baseline | Control of confounding factors | Assessment of outcome | Adequacy of follow-up | Consistency |  |
| Dupuis 2020 | 1 | 1 | 1 | 1 | 2 | 1 | 1 | 0 | 8 |
| Kim 2021 | 1 | 1 | 1 | 1 | 1 | 1 | 0 | 0 | 6 |
| Oh 2020 | 0 | 0 | 1 | 1 | 1 | 1 | 0 | 0 | 4 |
| Park 2015 | 0 | 1 | 1 | 1 | 2 | 1 | 1 | 1 | 8 |
| YOO 2017 | 0 | 0 | 1 | 1 | 1 | 1 | 1 | 1 | 6 |
| Korhonen 2023 | 1 | 1 | 1 | 1 | 1 | 1 | 1 | 1 | 8 |
| Mostofsky 2009 | 0 | 1 | 0 | 1 | 1 | 1 | 0 | 1 | 5 |
| Putaala 2011 | 0 | 0 | 1 | 1 | 1 | 1 | 1 | 1 | 6 |
| Chung 2025 | 1 | 1 | 1 | 1 | 2 | 1 | 1 | 1 | 9 |
| Penno 2020 | 1 | 0 | 1 | 1 | 2 | 1 | 1 | 1 | 8 |
| Moriya 2017 | 1 | 1 | 1 | 0 | 1 | 1 | 1 | 0 | 6 |

Table S3 Subgroup analysis

| **Outcome** | **Subgroup*** | **HR 95%CI** | **Number of studies** |
| --- | --- | --- | --- |
| All-cause mortality | General population | 1.22 [1.09; 1.36] | 3 |
| All-cause mortality | Diabetes | 1.44 [1.16; 1.80] | 1 |
| All-cause mortality | People with CVD risk | 1.50 [1.06; 2.11] | 3 |
| CVD | General population | 1.44 [0.91; 2.28] | 2 |
| CVD | Diabetes | 1.13 [1.11; 1.15] | 1 |
| All-cause mortality | MDRD | 1.72 [1.16; 2.55] | 2 |
| All-cause mortality | CKD-EPI | 1.27 [1.16; 1.40] | 5 |

*The general population comprised participants from community settings or national databases without specific disease history. People with CVD risk included those with stage 1 hypertension, dyslipidemia, transient ischemic attack, or first ischemic stroke.

MDRD: the Modification of Diet in Renal Disease equation; CKD-EPI: the Chronic Kidney Disease Epidemiology Collaboration equation.

Table S4 Sensitivity analyses for all-cause mortality

| **Omitting one study** | **HR 95%CI** |
| --- | --- |
| Kim2021 | 1.36 [1.21; 1.53] |
| Korhonen2023 | 1.30 [1.19; 1.43] |
| Mostofsky2009 | 1.29 [1.17; 1.41] |
| Park2015 | 1.28 [1.16; 1.42] |
| Penno2020 | 1.27 [1.14; 1.40] |
| Putaala2011 | 1.28 [1.17; 1.41] |
| YOO2017 | 1.31 [1.18; 1.45] |


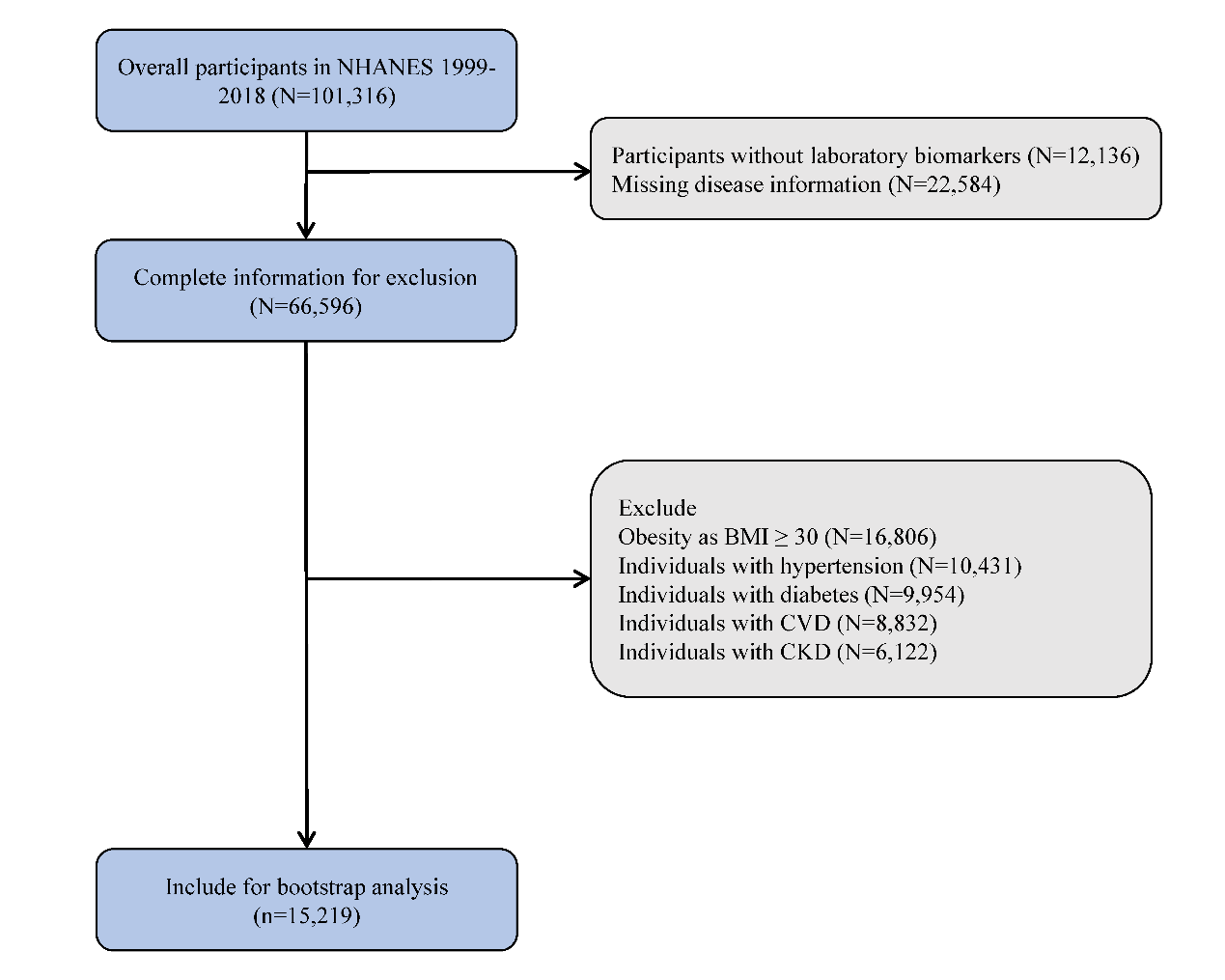


Figure S1. Flow chart of NHANES for bootstrap.
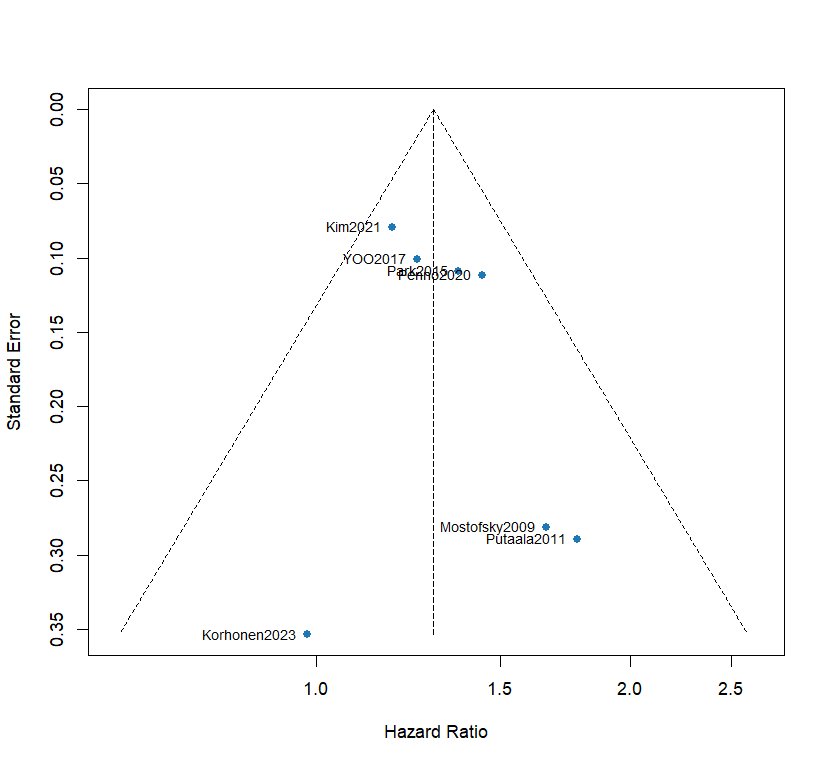


Figure S2. Funnel plot of mortality outcome to test publication bias


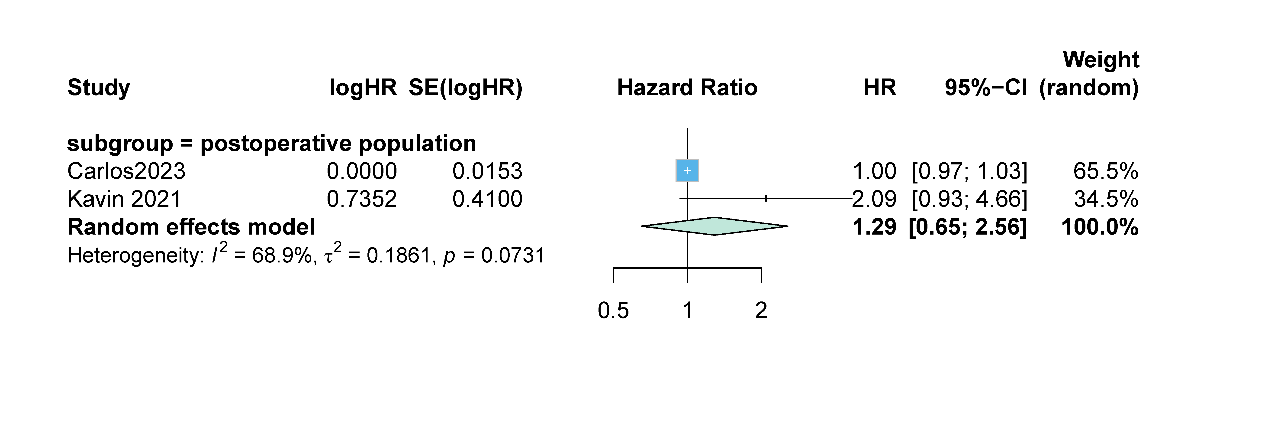
Figure S3 Meta-analysis between GHF and 30-day major adverse events


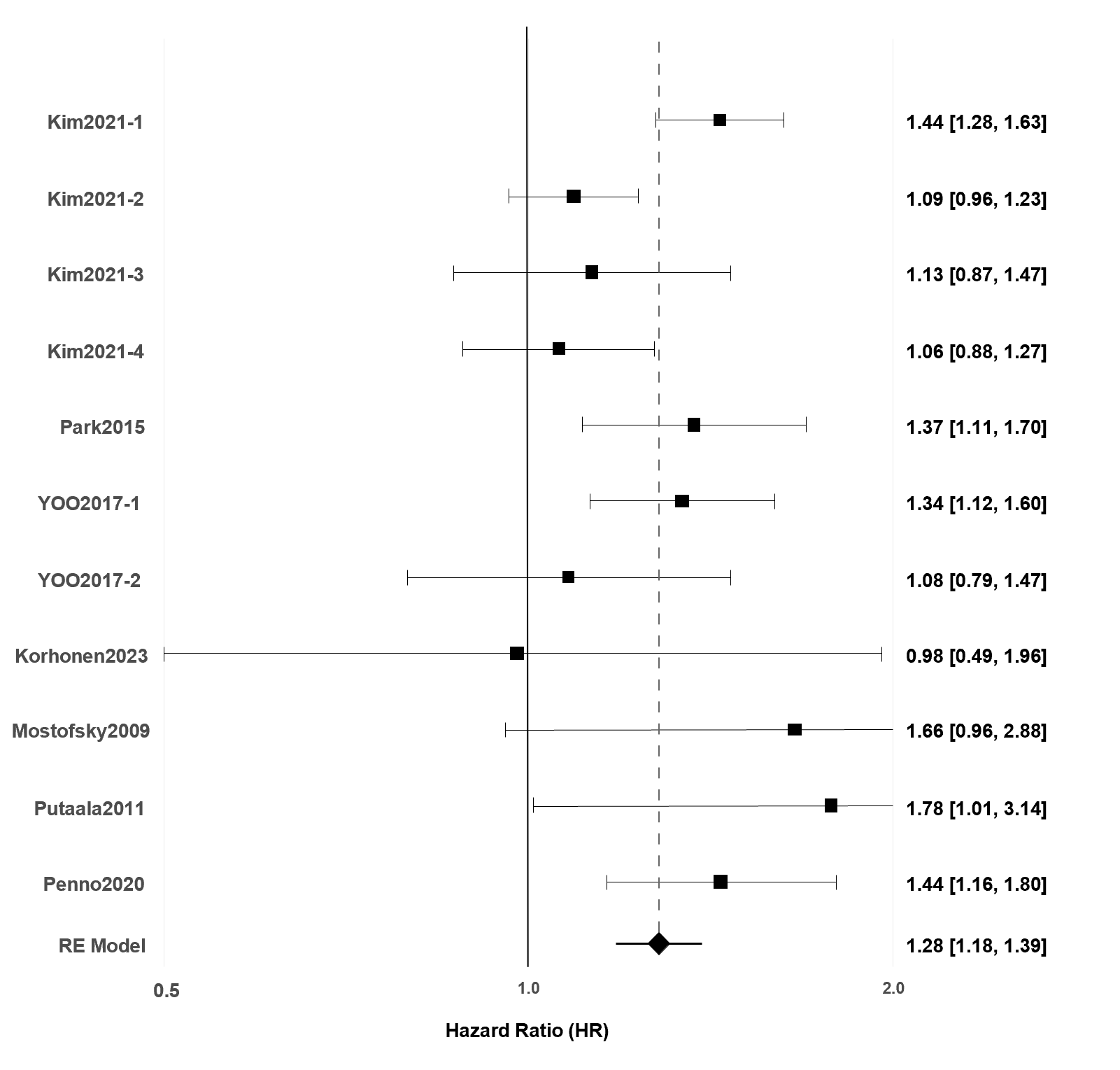


Figure S4 Multilevel random-effects models for all-cause mortality
